# Exploring Somatic Alteration Associating With Aggressive Behaviors of Papillary Thyroid Carcinomas by Targeted Sequencing

**DOI:** 10.3389/fonc.2021.722814

**Published:** 2021-10-07

**Authors:** Yi Li, Wei Gao, Xiaojun Cai, Anqi Jin, Jian Shen, Yichun Zhang, Yutong Chen, Bing Hu, Tao Zeng, Xiangtian Yu, Yuanyi Zheng, Yan Wang

**Affiliations:** ^1^ Department of Ultrasound in Medicine, Shanghai Jiao Tong University Affiliated Shanghai Sixth People’s Hospital, Shanghai, China; ^2^ Shanghai Institute of Ultrasound in Medicine, Shanghai, China; ^3^ Department of Ultrasound Medicine, Fudan University Shanghai Cancer Center, Shanghai, China; ^4^ Department of Oncology, Fudan University Shanghai Cancer Center, Shanghai, China; ^5^ CAS Key Laboratory of Computational Biology, Bio-Med Big Data Center, Shanghai Institute of Nutrition and Health, University of Chinese Academy of Sciences, Chinese Academy of Sciences, Shanghai, China; ^6^ Key Laboratory of Systems Biology, Shanghai Institute of Biochemistry and Cell Biology, Center for Excellence in Molecular Cell Science, Chinese Academy of Sciences, Shanghai, China; ^7^ Clinical Research Center, Shanghai Jiao Tong University Affiliated Sixth People’s Hospital, Shanghai, China

**Keywords:** prognosis, papillary thyroid carcinomas, targeted sequencing, risk stratification, gene test

## Abstract

Wisely differentiating high-risk papillary thyroid carcinoma (PTC) patients from low-risk PTC patients preoperatively is necessary when comes to making a personalized treatment plan. It is not easy to stratify the risk of patients according to sonography or lab results before surgery. This study aims to seek out potential mutation gene markers that may be helpful in stratifying the risk of PTC. A custom panel of 439 PTC relevant and classic tumor metabolic pathway relevant genes was designed. Targeted capture sequencing was performed on 35 pairs of samples from 35 PTC tumors and 35 para-tumor thyroid tissues obtained during surgery. Variant calling and detection of cancer gene mutations were identified by bio-information analysis. Ingenuity Pathway Analysis (IPA) was performed to do functional enrichment analysis of high-frequency mutant genes. Immunohistochemistry (IHC) was performed on 6 PTC patients to explore the expression of protein associated with interested genes. Event-free survival (EFS) was calculated to determine which genes might affect the prognosis of patients. We have identified 32 high-frequency mutant genes in PTC including *BRAF. RBL2* was found to be significantly correlated to event-free survival, *FOXO1*, *MUC6*, *PCDHB9*, *NOTCH1*, *FIZ1*, and *RTN1* were significantly associated with EFS, while BRAF mutant was not correlated to any of the prognosis indicators. Our findings in this study might open more choices when designing thyroid gene panels used in FNA samples to diagnose PTC and predict the potentially aggressive behavior of PTC.

## 1 Introduction

Thyroid carcinoma is the most common malignant tumor in the endocrine system which accounts for 96% malignant tumors of the head and neck. Most occurrences of thyroid carcinoma are papillary thyroid carcinoma (PTC) (1–4). Although overall prognosis of PTC is good and the mortality is stable at the rate of approximately 0.5 deaths per 100,000 (5, 6), clinical evidence has shown the performances of PTC patients are quite different (3, 7). On average, 50% of the patients are presented with lymph node metastases (LNM) at diagnosis, and in some cases, patients with micro papillary thyroid carcinoma could have macronodular lung metastasis (8, 9).

Considering the different nature of PTC and potential complication of thyroidectomy, doctors have to make a personalized treatment plan by wisely differentiating high-risk PTC patients from low-risk ones (10). Surgery is the most effective way to treat patients with PTC (11). Meanwhile, some other techniques are also applied gradually in PTC treatment, such as ultrasound-guided laser, radiofrequency, microwave ablation and percutaneous ethanol injection therapy (12–14). A strategy of active surveillance (AS) instead of immediate surgery is also proposed for confirmed low-risk PTC patients (15).

**Figure 1:**
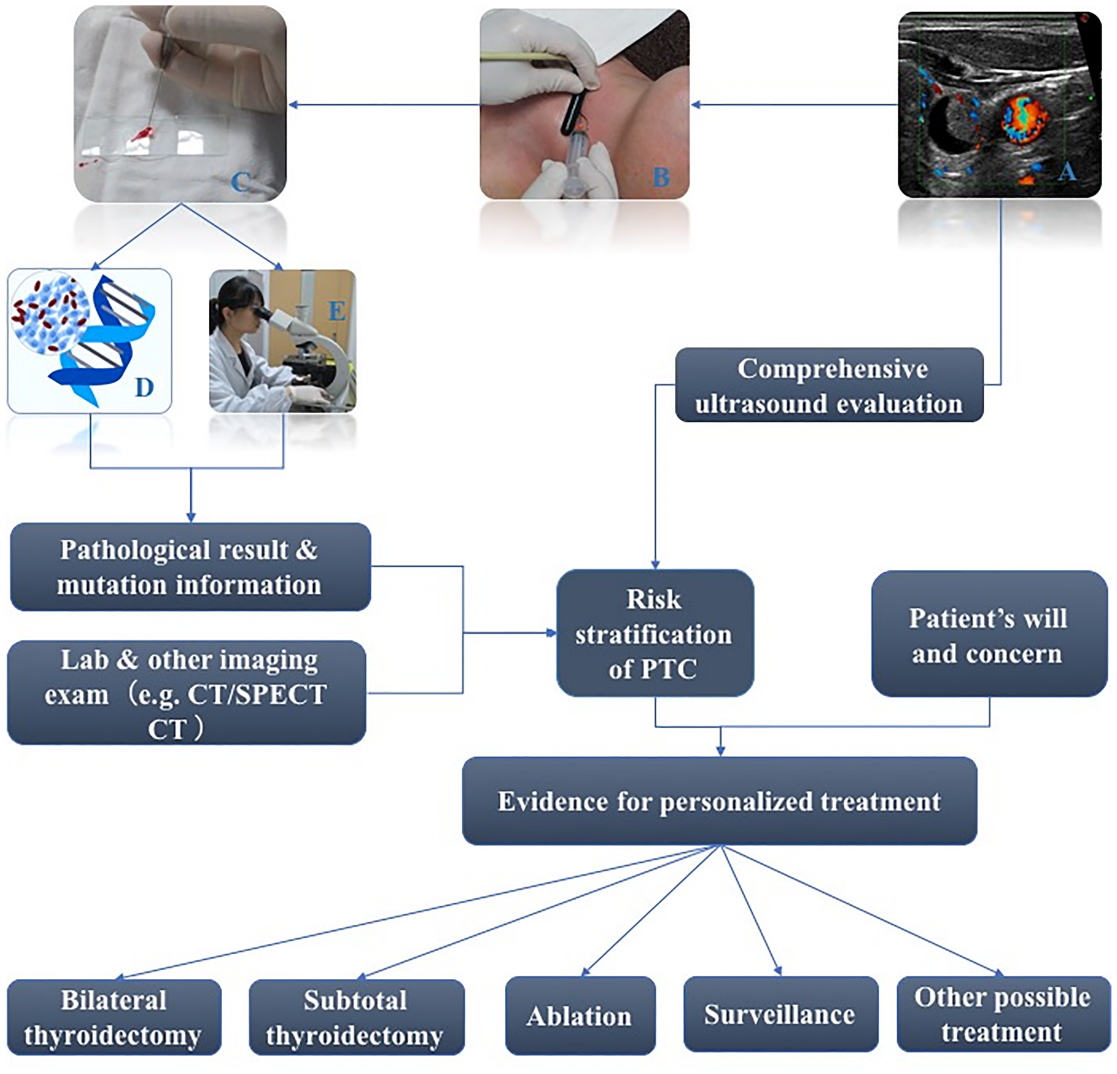
Workflow of FNA of thyroid nodule and how gene test participates in management of thyroid nodule (**A**: Sonography of a thyroid nodule ready for FNA; **B**: Real scene of performance of FNA; **C**: Preoperative FNA Smears; **D**: A figure of gene test; **E**: Traditional morphology diagnosis based on FNA Smears).

But how should high-risk PTC patients be wisely differentiated from low-risk PTC patients preoperatively? Over the years, multiple staging systems have been developed to predict the risk of mortality in patients with PTC. Each of the systems uses particular combinations of some clinical factors including age at diagnosis, size of the primary tumor, specific tumor histology, and extra-thyroidal spread of the tumor (direct extension of the tumor outside the thyroid gland, loco-regional metastases, and/or distant metastases), which tend to stratify patients into one of several categories with different risks of death from thyroid cancer (11, 16). Pre-operating TSH lever, TMN stage when diagnosed, and histology subtypes are also factors associated with PTC progression (5, 7). However, preoperative evaluation still has difficulties with demonstrating all of the clinical information because some of factors can only be confirmed by post-operation pathological results. Fine needle aspiration (FNA) is an efficient method for evaluating thyroid nodules that has high sensitivity and specificity, and gene testing based on FNA samples could be a potential tool to stratify the risk of PTC. The key problem for this possibility is which genes and types of the alteration should be chosen to identify for screening the high-risk patients with PTC. **Figure 1** demonstrates the workflow of FNA of the thyroid nodule and how gene testing participates in the management of the thyroid nodule.

Presently, next generation sequencing (NGS) technology provides the simultaneous analysis of hundreds of genes of interest, using targeted sequencing panels and NGS-based molecular tests performed on thyroid cancer to seek more valuable information to differentiate III/IV nodules from malignant ones (17–19). Currently, there is less evidence showing specific gene mutations reported for the recurrence or aggressive behaviors of PTC. Thus, in this study, a custom panel was designed and targeted sequencing would be performed to seek potential mutation gene markers helpful for stratifying the risk of PTC.

## 2 Methods

### 2.1 Patients and Samples

#### 2.1.1 General Information and Groups of Patients

The study included 41 PTC patients operated on in hospital between June 2016 and January 2017. 70 tissue samples including tumor tissue (n=35) and para-carcinoma tissue (n=35) samples were obtained during surgery and preserved in liquid nitrogen immediately and 6 paraffin-embedded samples obtained from other patients were sliced. All patients provided written informed consent for sample collection and analysis. The study protocol was approved by Shanghai 6^th^ people’s hospital institutional review board and adhered to the Declaration of Helsinki.

(i) Candidate samples for targeted NGS

The cohort of 35 patients in the study group was categorized into two groups: recurrence group (n=8) and no-recurrence group (n=27). The standard of recurrence was as below: new lymph nodes appearing confirmed as metastasized by cytological exam; New mass appearing around thyroid bed confirmed as metastasized by cytological exam; New lymph nodes with obvious malignant characteristics such as liquefaction or calcification with increased TG level; Distant metastasis; Obvious increase in TG without positive imaging findings but undergoing significant reduction after iodine therapy. Meeting any of these rules would be considered as recurrence. If the patient did not meet any of the rules during the following up period, it would be considered as no-recurrence.

Besides being grouped as recurrence or not, the patients were also regrouped into aggressive PTC group (n=18) and mild PTC group (n=17). The aggressive PTC group contained patients with extra-thyroidal invasion or severe lymph nodes involvement (≥5) at surgery or with recurrence while the mild group did not meet any of the aggressive behaviors mentioned above.

(ii) Candidates for Immunohistochemistry (IHC)

Samples of tumor tissue and normal thyroid tissue from 6 PTC patients in the assessment group which were prepared for validating the NGS findings were randomly selected among 318 patients who underwent thyroid surgery in our hospital. The information of the 6 patients was listed in **Table S1**. One patient died 3 years after their first surgery because of hemoptysis due to PTC recurrence. One patient was confirmed to have multiple lymph nodes metastasis months after surgery. One patient was confirmed to have bone metastasis during surgery. These three patients belonged to the high-risk assessment group, and the other three patients belonged to low-risk assessment group, which were considered relatively mild because of unilateral nodules, no metastatic lymph nodes, and no recurrence in 5 years of follow-up.

#### 2.1.2 Following Up

All of the patients were followed up and the last dates of follow-up were recorded on August 21^st^ 2020. The events of death, recurrence, or metastasis were recorded by follow-up secretary.

### 2.2 Targeted Capture Sequencing of PTC Genes

#### 2.2.1 Design of SureSelect Custom Panel of PTC Relevant Genes

We performed targeted capture sequencing of prior-collected PTC genes in 70 tissue samples including 35 tumor samples and 35 para-carcinoma tissues, as described previously.

We designed a SureSelect custom panel of PTC relevant genes (Agilent Technologies, Santa Clara, CA) (**Table S2**). The gene list in the panel includes genes recurrently mutated in PTC; genes mutated in stage IV PTC analyzed and extracted from TCGA and COSMIC database; genes closely related to the relevant biological pathways including cell cycle, cell growth and proliferation, survival/cell death regulation, TP53 signaling, Notch signaling, DNA Damage response, telomere maintenance, RTK signaling family, PI3K-AKT-mTOR signaling, RAS-RAF-MEK-ERK/JNK, regulation of ribosomal protein synthesis and cell growth angiogenesis folate transport invasion and metastasis; and genes mentioned in relative literature and commercial panel. There is 3.342 Mbp in each panel including 439 genes, 8541 regions and 109254 probes. Extracted genomic DNA was fragmented and bait-captured according to manufacturer protocols. Captured DNA libraries were then sequenced using a HiSeq 2000 sequencer (Illumina, San Diego, CA). Raw image files were processed by Illumina base-calling Software 1.7 for base-calling with default parameters and the sequences of each individual were generated as 90/100bp pair-end reads.

#### 2.2.2 Variant Calling and Detection of Genes With High-Frequency Somatic Mutation

Raw FASTQ data were generated from the Illumina pipeline, the adapter sequence in the raw data and low-quality reads were removed in the QC step, and the remaining “clean reads” were used for downstream bioinformatics analysis. Standard bioinformatics analysis was firstly performed on the clean data by Burrows-Wheeler Aligner (BWA) to do the sequence alignment. The human genome build37 (hg19) was used as the reference genome for this project which is available at http://hgdownload.cse.ucsc.edu/goldenPath/hg19/bigZips/. Picard (http://picard.sourceforge.net/) was used to fix mate information of the alignment, add read group information and remove duplicate reads caused by polymerase chain reaction (PCR). After these processes, the obtained BAM files were used to do the variant calling. Single Nucleotide Polymorphisms (SNPs) was obtained by GATK (http://www.broadinstitute.org/gsa/wiki), and annotated on the basis of known protein-coding and non-protein-coding genes taken from the NCBI RNA reference sequences collection (RefSeq). To remove false-positive mutations, these SNPs were validation and compared in databases like dbSNP (The Single Nucleotide Polymorphism Database) and 1000 Genomes (The 1000 Genomes Project), and their functionality and conservation annotation were carried out according to SIFT, PolyPhen-2, PhyloP, etc. Meantime, the InDels were also called and annotated as the similar pipeline. Somatic Single Nucleotide Variants (SNVs) and Small Insertion/Deletion (InDels) were detected by Varscan (20) and GATK, which identified the tumor specific somatic substitutions by comparing PTC tumor and normal para-carcinoma in a pair. Then they were given functional annotation and classification by ANNOVAR in the above similar filtering pipeline. QualByDepth (QD), FisherStrand (FS), RMSMappingQuality (MQ), MappingQualityRankSum (MQRankSum) and ReadPosRankSum were applied to filter out variants with low quality. Only variants which would cause an obvious protein-coding change were kept, where specifically, the variants with an ANNOVAR annotation of missense_variant, splice_acceptor_variant, splice_donor_variant, splice_region_variant, start_lost,stop_gained, stop_lost, initiator_codon_variant, were counted. Mutation plots were generated using R package complexHeatmap (21).

#### 2.2.3 Functional Enrichment Analysis of Genes With High-Frequency Somatic Mutation by Ingenuity Pathway Analysis

We captured the high-frequency mutant genes of PTC through counting the mutation frequency of the genes with protein-coding change SNVs in all patients. These genes are generally thought to be related to the pathogenicity of thyroid cancer. To further uncover potential disease-associated biological functions regulated by these candidate driver genes, we analyzed these genes by IPA database with diverse functional annotation and enrichment analysis (22).

#### 2.2.4 Mutant Genes With High-Frequency Somatic Mutation Associated to Aggressive Behaviors of PTC

Gene mutation frequency was presented as frequencies or percentages, Fisher’s exact test was carried out between “mild PTC” and “aggressive PTC” to search the mutant genes associated with high-risk progression of PTC patients, where statistical significance was defined as one-sided values of p<0.05.

To guarantee clinically detectable/observable protein expression of these mutant genes in thyroid tissue, these candidates were all screened in THE HUMAN PROTEIN ATLAS database (23), which can help provide efficient targets for Immunohistochemistry (IHC) validation of potential protein expression/function change involved in PTC (24).

#### 2.2.5 Immunohistochemistry

Samples of 6 PTC patients were taken to detect the potential protein expression/function alterations of final gene candidates we found, where the 6 patients of IHC assessment group were independent from 35 patients of study group mentioned before (**Table S1**). The catalogs of the antibodies for the 5 proteins are (BS71182 from Bioworld, 101107-T10 from Yiqiao Shenzhou, Ab189647 from Abcam and CY5319 from Abcam).

The samples were fixed in 4% buffered paraformaldehyde, decalcified in 50 mM EDTA, embedded in paraffin, and sectioned at 5-mm thickness. Samples were deparaffinized and rehydrated. Twelve high-power fields were randomly selected and photographed in each sample, and the percentage of positive area was used to evaluate coding proteins of target genes. Immunochemistry was used to evaluate protein expressions of candidate mutant genes. The average optical density also called IOD (integrated optical density/area) of positive reactions was analyzed with Image Pro Plus 6.0 software.

### 2.3 Exploration of the Potential Prognostic Mutant Gene

For each PTC patient, the event-free survival (EFS) was defined as the time between surgical treatment and recurrence. Survival curves were plotted with the Kaplan-Meier method, and differences between mutant and non-mutant groups were compared by the log-rank test. Statistical significance was defined as two-sided values of P < 0.05.

### 2.4 Statistics

Clinical data was presented either as frequencies and percentages, or as means and standard deviations, or medians and interquartile range. Categorical variables were compared using either Pearson chi-square test or Fisher’s exact test. Continuous variables were compared using either the independent t test or one-way analysis of variance. Statistical significance was defined as two-sided values of P < 0.05. Of note, as limited number of samples and targeted sequencing were used in this study, the (high) mutation frequency instead of multitest correction was applied to further control the potential false-positive.

**Figure 2:**
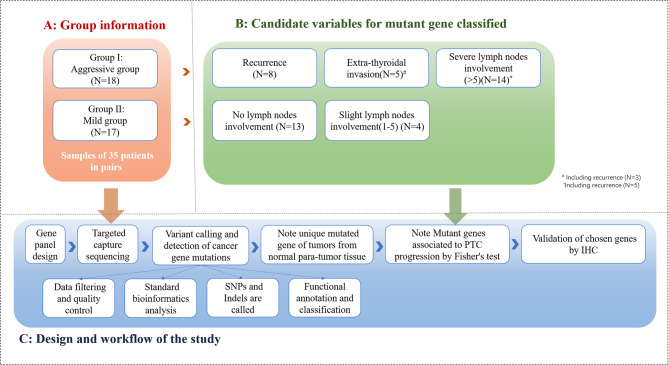
Method and flow of the study (The cohort of 35 patients in study group was categorized into 2 groups: PTC group (n=18) and mild PTC group (n=17). Aggressive PTC contains 8 patients with recurrence, 5 patients with extra-thyroidal invasion, 14 patients with severe lymph nodes involvement; Mild PTC group contains 13 patients without lymph nodes involvement and 4 patients with 0-5 lymph nodes involvement. 70 samples from 35 paired of patients were taken into NGS and bio-information analysis).

Statistical analyses were conducted with SPSS version 23.0 (SPSS Inc). **Figure 2** demonstrates the method and flow of the study.

### 2.5 Code and Data

The raw data generated for this study can be found in The National Omics Data Encyclopedia (NODE), project ID OEP002494. The related analysis and visualization scripts and supported data can be accessed at https://github.com/graceyu1985/TargetedPTC.

## 3 Results

### 3.1 Patient Characteristics

Our study group cohort underwent NGS consisting of 35 patients diagnosed with classic PTC. The median age was 43.9 years, ranging from 15 to 80 years, and these patients were predominantly female (60%, 21/35). The median PTC tumor size was 1.8 cm, ranging from 0.3 to 5.0 cm. 62.9%(22/35) of the patients underwent total thyroidectomy, 54.3%(19/35) of the patients underwent unilateral or bilateral central part lymph node dissection while 45.7% (16/35) of the patients got central & lateral cervical lymph node dissection. After surgery, 28.6% (10/35) of the patients got iodine RAI therapy of which 5 patients did this for prophylactic treatment. Patients were staged using the eighth edition of the American Joint Cancer Committee/Union for International Cancer Control (AJCC/UICC) staging system. Also, patients’ recurrence risk was stratified by ATA guidelines (11). One patient died of hemoptysis in the second year of following up. Another 7 patients were confirmed to have recurrence during the 4-year following up. 27 patients did not show any ultrasonographic evidence or lab results indicating recurrence. The patients were divided into two groups according to whether they recurred, no less than 5 metastatic lymph nodes confirmed after the first surgery, the preoperative ATA risk classification and RAI therapy was related to recurrence(p=0.031, 0.012 and 0.048). Tumor size, age, gender, extra thyroidal invasion, BRAFv600E mutation, multifocality, and TMN Stage did not show significant relation to recurrence (**Table 1**).

**Table 1:** Clinical characteristic of patients with PTC in study groups.

	Recurrence n (%)	no report of recurrence n (%)	p Value
Number of patients	8 (22.86)	27 (77.14)	0.074
Age	35.13 (15-68)	46.44 (16-80)	
Gender
male	2 (5.71)	12 (34.29)	0.431
female	6 (17.14)	15 (42.86)	
Tumor size(mm)	2.08 (0.6-5.0)	1.72 (0.3-5.0)	0.481
Microcarcinoma
Yes	2 (5.71)	10 (28.57)	0.429
no	6 (17.14)	17 (48.57)	
Extra thyroidal invasion
yes	3 (8.57)	2 (5.71)	0.067
no	5 (14.29)	25 (71.43)	
Vascular infiltration
yes	1 (2.86)	0 (0)	0.229
no	7 (20.00)	27 (77.14)	
multifocality
yes	5 (14.29)	14 (40.00)	0.452
no	3(8.57)	13 (37.14)	
LN involvement (>5)
yes	6 (17.14)	9 (25.71)	0.031
no	2 (5.71)	18 (51.43)	
BRAFv600E mutation			
Yes	5 (14.29)	16 (45.71)	0.602
no	3 (8.57)	11 (31.43)	
ATA Risk evaluation
HIGH	2 (5.71)	0 (0)	0.012
INTERMEDIATE	4 (11.43)	10 (28.57)	
LOW	2 (5.71)	17(48.57)	
TMN Stage
I	6 (17.14)	22 (62.86)	0.268
II	0 (0)	5 (14.29)	
III	2 (5.71)	0 (0)	
Operation type
Total thyroidectomy	6 (17.14)	16 (45.71)	0.695
subtotal thyroidectomy	2 (5.71)	11 (31.43)	
Lymphadenectomy
Unilateral central	2 (5.71)	13(37.14)	0.142
bilateral central	0 (0)	4 (11.43)	
Central+ Lateral cervical	6 (17.14)	10 (28.57)	
RAI therapy
Yes	5 (14.29)	5 (14.29)	0.048
No	3 (8.57)	22 (62.86)	

### 3.2 Mutation Profiling of Patients With PTC

**Figure 3:**
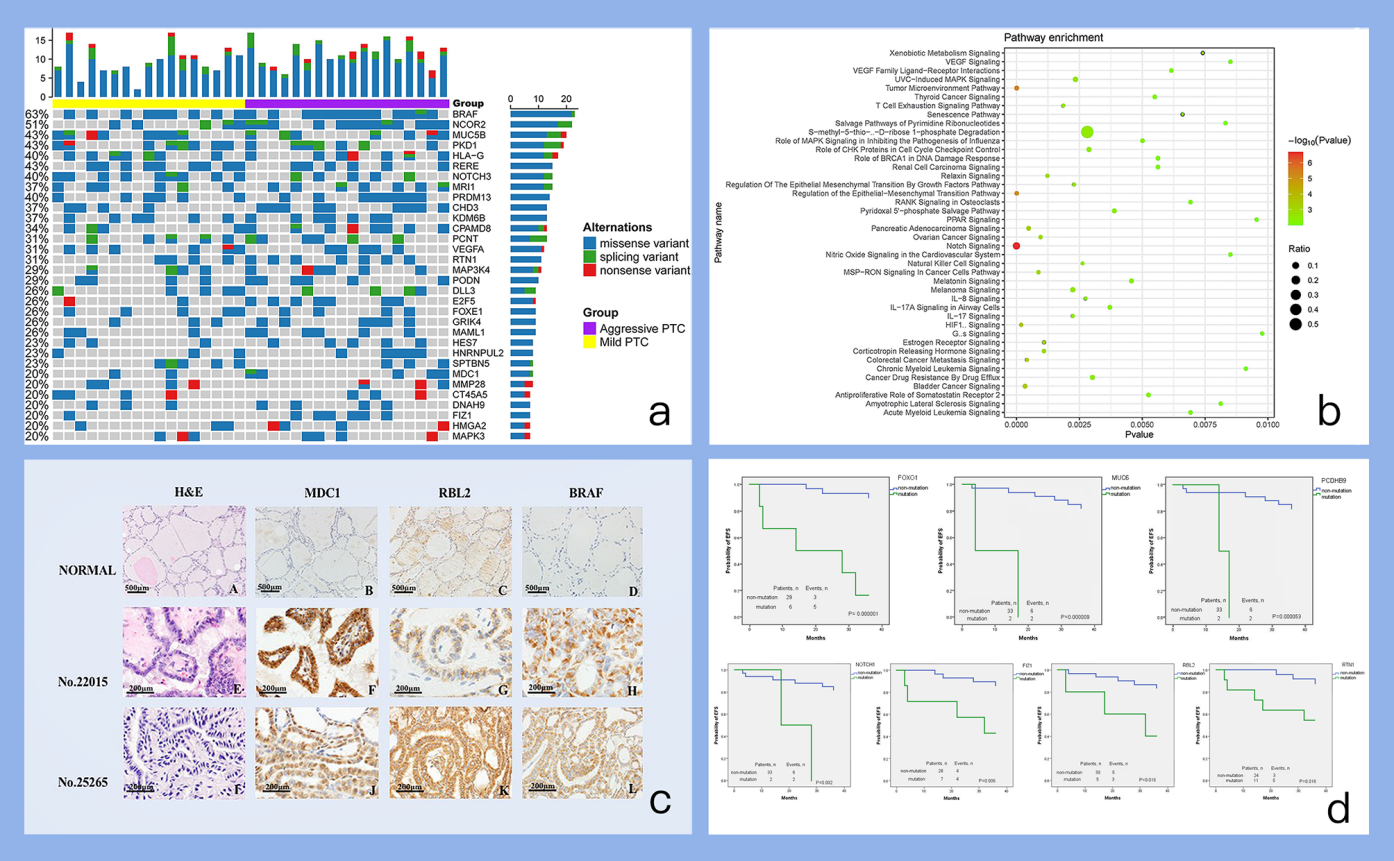
Figures of important findings. **(a)** Mutation profile of high-frequency somatic mutant genes. Each row represents a somatic mutant gene, and each column represents one individual. The bar on each row indicates the mutation number (mutation frequency) of one gene occurring on all individuals. The bar on each column indicates the number of mutated genes for one individual. **(b)** Pathway enrichments of high-frequency somatic mutant genes. The pathways enriched with high-frequency somatic mutant genes are evaluated and identified by IPA tool. **(c)** IHC results of candidate mutant genes. A-D. Normal thyroid tissue with HE, MDC1, RBL2, BRAF IHC staining. E-H. PTC tumor sample (No.22015) from patients confirmed recurrence with HE, MDC1, RBL2, BRAF IHC staining. I-L PTC tumor sample (No.25265) from patients without confirmed recurrence with HE, MDC1, RBL2, BRAF IHC staining). **(d)** Survival analysis of selected candidate mutant genes. Each subplot displays the survival plot of one mutant gene candidate.

We analyzed 439 genes including 8541 regions and 109254 probes using the panel on matched tissue samples from 35 PTC patients. The statistical results are shown in **Figure 3a** and **Table S3**. The average sequencing depth of the target region is about 367.77 X. on average, 99.82% of the target region is covered by at least one read, and 99.12% of the target region is covered by at least 10 reads. Mutations were identified in 35 (100%) PTCs, of which *BRAF* (62.86%) and *NCOR2* (51.43%) mutations were exceeded among 50% patients. Multiple high-frequency mutant genes (with mutations in >20% patients) were found in PTCs by the NGS assay and further analysis is required to reveal PTC-associated biological functions regulated by these mutant genes.

We analyzed 32 high-frequency mutant genes (mutant in >20% patients) by IPA (Ingenuity Pathway Analysis) database with diverse functional annotation and enrichment analysis. The results from IPA analysis (**Table S4**) also supplied many other functional evidences for our selected genes. For example, in the pathway enrichment analysis results (**Figure 3b**), “Notch Signaling” pathway is significantly enriched and mutant genes can participate in many novel pathways such as: “Regulation of the Epithelial-Mesenchymal Transition Pathway”, and “Tumor Microenvironment Pathway”.

Of note, we have also summarized the mutation frequency of another PTC driver gene *RAS* as shown in **Figure S1**. Actually, *RAS* tends to have a remarkably high mutation frequency, and especially have more mutations in “aggressive PTC” than those in “mild PTC”. However, the dominant mutation type annotated of *RAS* was “intron variant” in our data. Thus, other PTC driver genes listed in **Figure 3c** were considered in the following analysis and discussion.

### 3.3 Genetic Comparison of “Mild PTC” and “Aggressive PTC”

We further examined the gene mutations in the subgroups “mild PTC” (i.e.17 patients from study group) and “aggressive PTC” (i.e. 18 patients from the study group). We carried out fisher’s exact test analysis between “mild PTC” and “aggressive PTC” to search the differentially mutated genes associated with PTC aggressive behaviors. The genes with one-sided values of P < 0.05 were final candidates. We found different altered genes through different group methods, and the genes are listed in **Table 2**. After being screened in THE HUMAN PROTEIN ATLAS database, we finally confirmed several candidates including “*MDC1*, *NCOR2*, *RBL2*” as well as *BRAF* for the following IHC experiment to further confirm their protein expression state and change during PTC occurrence and progression. Of note, we have also tried to build a logistic regression (LR) model based on these mutant genes for predicting risk classification. The LR model was built on 70% of samples, and evaluated to predict the remaining 30% of samples for belonging to “mild PTC” or “aggressive PTC”, which achieved AUC=0.64 and ci= [0.33,0.95] (**Figure S2**).

**Table 2:** List of aggressive PTC special mutated genes by fisher’s test in different groups.

Recurrence		More than 5 metastatic lymph nodes		Extra-thyroidal invasion	
Gene name	p value	Gene name	p value	Gene name	p value
NOTCH1	0.047	HNRNPUL2	0.067	MDC1	0.090
MUC6	0.047	GRIK4	0.010	EGFR	0.069
FOXO1	0.001	FOXO1	0.026	NCOR2	0.015
RTN1	0.077	PRDM13	0.006	ITGAX	0.056
RBL2	0.067	FOXE1	0.095	RBL2	0.006
DLL3	0.081			FIZ1	0.090
FIZ1	0.033			PDGFB	0.069
PCDHB9	0.047				

### 3.4 Protein Expression of Mutant Gene Candidates in PTC

Immunochemistry was used to evaluate protein expression of *MDC1*, NCOR2, *RBL2*, and *BRAF*, which can provide additional complementary clinical assessment of the functional protein loss of these coding genes (25). According to the IHC results, all of these candidates have higher protein expressions in tumors no matter their categorization as mild cases or aggressive cases than those in normal thyroid tissue (**Figure 3c** and **Table S5**). Particularly, the average expression of MDC1 protein was higher in aggressive cases than those in mild cases (0.434 ± 0.057 vs 0.177 ± 0.021). And the average expression of RBL2 protein was lower in aggressive cases than those in mild cases (0.165 ± 0.016 vs 0.371 ± 0.054).

### 3.5 Prognostic Mutant Gene for PTC

In addition, the prognostic potential of those four mutant gene candidates were also evaluated by Kaplan-Meier method. *RBL2* was found to be remarkably related to event-free survival (P=0.016). Considering many potential functional contributions except for protein expression would also be caused by mutation, other differentially mutated genes associated with PTC disease progression were re-screened again, and it was actually found that *FOXO1*, *MUC6*, *PCDHB9*, *NOTCH1*, *FIZ1*, *RTN1* could be significantly associated with EFS (**Figure 3d**).

## 4 Discussion and Conclusion

Several studies have applied NGS on PTCs (3, 9, 26, 27). The Cancer Genome Atlas (TCGA) Network described the genomic characterization of 496 PTCs, and generated data using whole genome sequencing. The TCGA project identified the TERT promoter mutation, which accounts for approximately 1% of PTCs, but shows association with a high risk of recurrence (19, 27). Nikiforova et al. applied the ThyroSeq series NGS panel targeting 12 genes or 14 genes to differentiate FNA III/IV nodules (18, 28). Picarsic et al. analyzed 17 pediatric PTCs by using ThyroSeq v2 panel and found ETV6-NTRK3 fusion was associated with aggressive histologic features. In this study, we focused on core genes related to aggressive behavior of PTC. We not only use recurrence as an indicator to select genes, but also considered severe lymph nodes involvement and extrathyroidal invasion as high risk indicators based on the evidence in the 2015 American Thyroid Association (ATA) management guidelines for adult patients with thyroid nodules and differentiated thyroid cancer (11). In this guideline, it was said that neck lymph node (LN) metastases are found in up to 70% of cases of PTC, and initial LN metastases as well as extra-thyroid infiltration have also been identified as an independent risk factor for distant metastases (29, 30). So, we considered all of the high-risk factors and found that *RBL2* was significantly correlated to event-free survival, *FOXO1*, *MUC6*, *PCDHB9*, *NOTCH1*, *FIZ1*, *RTN1* could be significantly associated with EFS, while *BRAF* mutant was not correlated to any of the prognosis indicators.

BRAFV600E mutation is considered as the most common mutation in PTC, but its role in predicting prognosis is unclear (31, 32). Recent data suggest that specific molecular profiles, such as the coexistence of *BRAF* with other oncogenic mutations (such as *PIK3CA*, *AKT1*), *TERT* promoter, or *TP53* mutations, may serve as more specific markers of less favorable outcomes of PTC (11). In our study, BRAFV600E showed a high mutation rate (62.86%) in the 35 patients, but it did not show any differentiating capability in picking cases of “aggressive PTC”. Other oncogenic mutations including *TERT* promoter and *TP53* mutations also did not show differentiating capability either. Another interesting finding is that *RAS* tend to have a remarkably high mutation frequency, and especially have more mutations in “aggressive PTC” than those in “mild PTC”. However, the dominant mutation type annotated of *RAS* was “intron variant” in our data. PTCs were separated into two groups of distinct downstream signaling pathways: the BRAFV600E-like cohort and the RAS-like cohort and most of the RAS-like PTCs were follicular variant PTCs (FVPTCs) (19, 27). In our cohort, there are all classic PTCs, it might help explain why meaningful RAS mutation was rare in our results. So, we did not do further bio-informative analysis in the RAS mutation pathway.

In this study, we have detected some mutant gene related protein candidates to explore the potential influence of these gene signatures, and successfully found that the expression of *MDC1* and *RBL2* proteins were different in aggressive cases than mild cases.

Rbl2(RB transcriptional corepressor like 2) belongs to the retinoblastoma (Rb) family which are considered tumor suppressors (33). RB is closely related to two genes in mice and humans (p107 and 130, which are not commonly mutated in tumors) (34). The retinoblastoma protein (RB) has been implicated in many cellular processes, such as regulation of the cell cycle, DNA-damage responses, DNA repair, DNA replication, protection against apoptosis, and differentiation. They could contribute to the function of RB as a tumor suppressor. The Rb can inhibit cell cycle progression through disabling the E2F family of cell-cycle-promoting transcription factors (35). In our study, we found *RBL2* mutated in tumor samples of the group confirmed as recurrence and its protein also called Rbl2 expressed in normal thyroid samples and PTC samples. However, the expression level was lower in patients with recurrence than in tumor samples belonging to patients without recurrence. There were few articles discussing the role of RB in the PTC driving process, and our study was the first to show a potential relation between RB and the prognostic results of thyroid cancer, which deserves further study.

Mediator of DNA damage checkpoint 1 (*MDC1*) is a scaffold protein involved in the early steps of DDR functioning as a tumor suppressor (36, 37). The DNA damage response (DDR) is necessary to maintain genome integrity and prevent the accumulation of oncogenic mutations (38, 39). Consequently, proteins involved in the DDR often serve as tumor suppressors, carrying out the crucial task of keeping DNA fidelity intact (40). Studies have found a correlation between *MDC1* loss and tumor aggression (41). In breast cancer and pancreatic cancer, low *MDC1* expression correlates with more invasive, later stage tumors, indicating that *MDC1* expression may serve to limit the progression of disease (39, 42–44). By contrast, it was found that MDC1 is upregulated in most cervical cancer samples (45), laryngeal squamous cell carcinoma samples (SCC) (46), and nasopharyngeal carcinoma samples (47, 48). Since SCC are usually driven by human papillomavirus infection (HPV), and nasopharyngeal carcinoma by Epstein-Barr virus (EBV), this made it possible to speculate that viral carcinogenesis activates the DNA damage response *via* several mechanisms (39, 48). In this study, we observed mutation of *MDC1* in the aggressive group, which indicates that the mutation might relate to more aggressive behavior. There are several studies that have reported a correlation between HBV and thyroid cancer (49, 50). And we might also boldly make a hypothesis that virus and viral carcinogenesis could play an important role in the drive and progression of PTC mediated by *MDC1* with inducing epithelial-mesenchymal-transition. The study of *MDC1* and thyroid cancer is few, and our results just unveiled the tip of the iceberg of its relations. This association between *MDC1* expression and its potentially oncogenic and tumor suppressive (dual) roles indicate an opportunity for further research such as a 3D tumor microenvironment on thyroid tumor development to understand its complex role in thyroid cancer (39, 51).

Of course, there are still several limits in the study. The numbers of samples were only 35 pairs which was relatively small to capture more mutant gene candidates. It is a single institute investigation using one classic type of PTC rather than all types of thyroid cancers so the sample size was relative small. The resulting data would still be insufficient for making decisions on either patient diagnosis or treatment in clinical practice. To overcome such limitations, an expansion of the study cohort with significantly increased patient sample size in our future work should help investigate and validate more and more new clinical findings on the basis of next-generation sequencing and immunohistochemistry technologies, such as building a more accurate and robust LR model for predicting risk classification.

As we discussed above, scholars have applied multiple panels in helping characterize III/IV nodules in FNA but not specified on aggressive PTCs. Future studies are expected to establish the impact of molecular profiling involving multiple mutations on clinical management of patients with a PTC smaller than 1 cm. Furthermore, characterization of tumor mutation profiles (e.g. exon sequencing) combined with US scan and laboratory results (e.g. immunohistochemistry) might also show a more comprehensive picture of a malignant thyroid nodule and it will also open more choices for therapeutic plans for PTC patients.

## Data Availability Statement

The datasets presented in this study can be found in online repositories. The names of the repository/repositories and accession number(s) can be found below: National Omics Data Encyclopedia (NODE) [accession: OEP002494].

## Ethics Statement

The studies involving human participants were reviewed and approved by Shanghai 6th people’s hospital institutional review board. Written informed consent to participate in this study was provided by the participants’ legal guardian/next of kin.

## Author Contributions

YL, WG, and XC contributed equally to this work. YCZ and YC obtained tumor and para-carcinoma tissue samples and completed follow-up. AJ performed clinic data statistical analysis. XC performed targeted capture sequencing of PTC genes. YL edited the manuscript. WG modified this article. YYZ and YW designed experiments. BH was the consultant, and supervised the project. XY and TZ performed Bioinformatics Analysis. All authors contributed to the article and approved the submitted version.

## Funding

This study was supported by the National Natural Science Foundation of China (No. 81701706, 61803360, 11871456), and the Shanghai Municipal Science and Technology Major Project (Grant No. 2017SHZDZX01).

## Conflict of Interest

The authors declare that the research was conducted in the absence of any commercial or financial relationships that could be construed as a potential conflict of interest.

## Publisher’s Note

All claims expressed in this article are solely those of the authors and do not necessarily represent those of their affiliated organizations, or those of the publisher, the editors and the reviewers. Any product that may be evaluated in this article, or claim that may be made by its manufacturer, is not guaranteed or endorsed by the publisher.

## Supplementary Material

Supplementary Figure 1 Mutation profile of RAS genes. Each row represents a RAS gene, and each column represents one individual. The bar on each row indicates the mutation number (mutation frequency) of RAS occurred on all individuals. The bar on each column indicates mutation number of RAS for one individual.

Supplementary Figure 2 ROC of logistic regression model for discriminating mild PTC and aggressive PTC groups.
