# Supplementary figures and images for: Exploring Somatic Alteration Associating With Aggressive Behaviors of Papillary Thyroid Carcinomas by Targeted Sequencing
Source: Front Oncol. 2021 Oct 7;11:722814. doi: 10.3389/fonc.2021.722814 (PMC8529196; doi:10.3389/fonc.2021.722814)

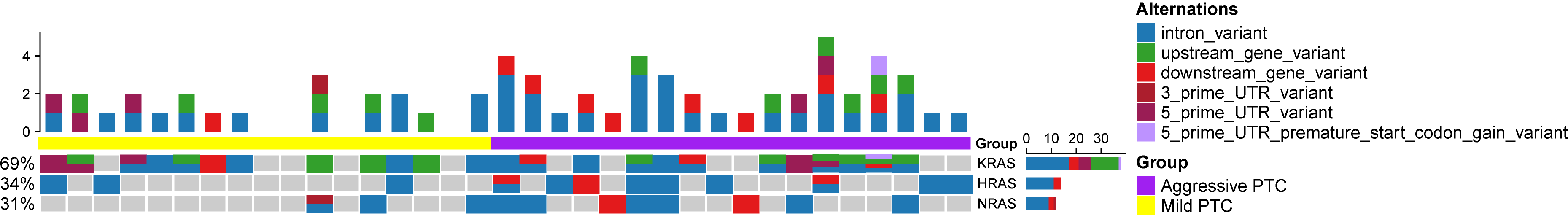

Supplement: Supplementary Figure 1 — Mutation profile of RAS genes. Each row represents a RAS gene, and each column represents one individual. The bar on each row indicates the mutation number (mutation frequency) of RAS occurred on all individuals. The bar on each column indicates mutation number of RAS for one individual. [file Image_1.tif]

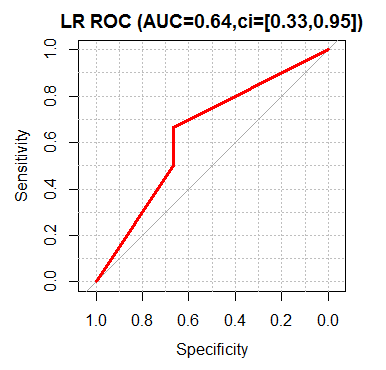

Supplement: Supplementary Figure 2 — ROC of logistic regression model for discriminating mild PTC and aggressive PTC groups. [file Image_2.tiff]
