# Supplementary material for: Exploring Somatic Alteration Associating With Aggressive Behaviors of Papillary Thyroid Carcinomas by Targeted Sequencing
Source: Front Oncol. 2021 Oct 7;11:722814. doi: 10.3389/fonc.2021.722814 (PMC8529196; doi:10.3389/fonc.2021.722814)
Supplement: Supplementary file 3 [file Table_1.docx]

Table S1 Clinical information of patients with PTC in IHC assessment group

|  | ID | gender | age | size（cm） | LN | Extrathyroidal invasion and distant metastasis | TMN stage | ATA risk Stratification | recurrence |
| --- | --- | --- | --- | --- | --- | --- | --- | --- | --- |
| Aggressive group | 2015-13819 | female | 22 | 4 | 17 | bone | II | HIGH | no |
|  | 2015-22015 | male | 37 | 3 | 10 | Fat tissue | I | HIGH | lymph nodes metastasis |
|  | 2015-40646 | male | 71 | 1.4 | 15 | capsule | II | INTERMEDIATE | died |
| Mild group | 2016-26179 | female | 60 | 0.5 | 5 | no | II | INTERMEDIATE | no |
|  | 2015-25265 | female | 66 | 1.2 | 0 | no | I | LOW | no |
|  | 2016-17171 | female | 49 | 2 | 0 | no | I | LOW | no |
