# Supplementary material for: Exploring Somatic Alteration Associating With Aggressive Behaviors of Papillary Thyroid Carcinomas by Targeted Sequencing
Source: Front Oncol. 2021 Oct 7;11:722814. doi: 10.3389/fonc.2021.722814 (PMC8529196; doi:10.3389/fonc.2021.722814)
Supplement: Supplementary file 4 [file Table_2.docx]

**Table S2 List of prior-collected PTC genes for targeted capture sequencing**

| **Categories** | **Member genes** |
| --- | --- |
| cell cycle control | RB1,RBL1,RBL2,CCNA1,CCNB1,CDK1,CCNE1,CDK2,CDC25A,CCND1,CDK4,CDK6,CCND2,CDKN2A,CDKN2B,MYC,CDKN1A,CDKN1B,E2F1,E2F2,E2F3,E2F4,E2F5,E2F6,E2F7,E2F8,SRC,JAK1,JAK2,STAT1,STAT2,STAT3,STAT5A,STAT5B |
| TP53 signaling | TP53,MDM2,MDM4,CDKN2A,CDKN2B,TP53BP1 |
| Notch signaling | ADAM10,ADAM17,APH1A,APH1B,ARRDC1,CIR1,CTBP1,CTBP2,CUL1,DLL1,DLL3,DLL4,DTX1,DTX2,DTX3,DTX3L,DTX4,EP300,FBXW7,HDAC1,HDAC2,HES1,HES5,HEYL,ITCH,JAG1,JAG2,KDM5A,LFNG,MAML1,MAML2,MAML3,MFNG,NCOR2,NCSTN,NOTCH1,NOTCH2,NOTCH3,NOTCH4,NRARP,NUMB,NUMBL,PSEN1,PSEN2,PSENEN,RBPJ,RBPJL,RFNG,SNW1,SPEN,HES2,HES4,HES7,HEY1,HEY2 |
| DNA Damge response 12 | CHEK1,CHEK2,RAD51,BRCA1,BRCA2,MLH1,MSH2,ATM,ATR,MDC1,PARP1,FANCF |
| other growth and proliferation | CSF1,CSF1R,IGF1,IGF1R,FGF1,FGFR1,AURKA,DLEC1,PLAGL1,OPCML,DPH1 |
| Survival/ cell death regulation 23 | NFKB1,NFKB2,CHUK,DIRAS3,FAS,HLA-G,BAD,BCL2,BCL2L1,APAF1,CASP9,CASP8,CASP10,CASP3,CASP6,CASP7,GSK3B,ARL11,WWOX,PEG3,TGFB1,TGFBR1,TGFBR2 |
| telomere maintenance | TERC,TERT |
| RTK signaling family | EGFR,ERBB2,ERBB3,ERBB4,PDGFA,PDGFB,PDGFRA,PDGFRB,KIT,FGF1,FGFR1,IGF1,IGF1R,VEGFA,VEGFB,KDR |
| PI3K-AKT-mTOR signaling | PIK3CA,PIK3R1,PIK3R2,PTEN,PDPK1,AKT1,AKT2,FOXO1,FOXO3,MTOR,RICTOR,TSC1,TSC2,RHEB,AKT1S1,RPTOR,MLST8 |
| RAS-RAF-MEK-ERK/JNK | KRAS,HRAS,BRAF,RAF1,MAP3K1,MAP3K2,MAP3K3,MAP3K4,MAP3K5,MAP2K1,MAP2K2,MAP2K3,MAP2K4,MAP2K5,MAPK1,MAPK3,MAPK4,MAPK6,MAPK7,MAPK8,MAPK9,MAPK12,MAPK14,DAB2,RASSF1,RAB25 |
| regulation of ribosomal protein synthesisi and cell growth | RPS6KA1,RPS6KA2,RPS6KB1,RPS6KB2,EIF5A2,EIF4E,EIF4EBP1,RPS6,HIF1A |
| angiogenesis | VEGFA,VEGFB,KDR,IL8,CXCR2,CXCR1 |
| floate transport | SLC19A1,FOLR1,FOLR2,FOLR3,FOLR4 |
| invasion and metastasis | MMP1,MMP2,MMP3,MMP7,MMP9,MMP10,MMP11,MMP12,MMP13,MMP14,MMP15,MMP16,MMP17,MMP19,MMP21,MMP23B,MMP24,MMP25,MMP26,MMP27,MMP28,ITGB3,ITGAV,PTK2,CDH1,SPARC,WFDC2 |
