# Supplementary material for: Exploring Somatic Alteration Associating With Aggressive Behaviors of Papillary Thyroid Carcinomas by Targeted Sequencing
Source: Front Oncol. 2021 Oct 7;11:722814. doi: 10.3389/fonc.2021.722814 (PMC8529196; doi:10.3389/fonc.2021.722814)
Supplement: Supplementary file 5 [file Table_3.docx]

Table S3 Summary of high-frequency mutant genes

| initial_alias | converted_alias | name | freq | percent | description |
| --- | --- | --- | --- | --- | --- |
| BRAF | ENSG00000157764 | BRAF | 22 | 62.86% | B-Raf proto-oncogene, serine/threonine kinase [Source:HGNC Symbol;Acc:HGNC:1097] |
| NCOR2 | ENSG00000196498 | NCOR2 | 18 | 51.43% | nuclear receptor corepressor 2 [Source:HGNC Symbol;Acc:HGNC:7673] |
| MUC5B | ENSG00000117983 | MUC5B | 15 | 42.86% | mucin 5B, oligomeric mucus/gel-forming [Source:HGNC Symbol;Acc:HGNC:7516] |
| PKD1 | ENSG00000008710 | PKD1 | 15 | 42.86% | polycystin 1, transient receptor potential channel interacting [Source:HGNC Symbol;Acc:HGNC:9008] |
| RERE | ENSG00000142599 | RERE | 15 | 42.86% | arginine-glutamic acid dipeptide repeats [Source:HGNC Symbol;Acc:HGNC:9965] |
| HLA-G | ENSG00000204632 | HLA-G | 14 | 40% | major histocompatibility complex, class I, G [Source:HGNC Symbol;Acc:HGNC:4964] |
| PRDM13 | ENSG00000112238 | PRDM13 | 14 | 40% | PR/SET domain 13 [Source:HGNC Symbol;Acc:HGNC:13998] |
| KDM6B | ENSG00000132510 | KDM6B | 13 | 37.14% | lysine demethylase 6B [Source:HGNC Symbol;Acc:HGNC:29012] |
| MRI1 | ENSG00000037757 | MRI1 | 13 | 37.14% | methylthioribose-1-phosphate isomerase 1 [Source:HGNC Symbol;Acc:HGNC:28469] |
| NOTCH3 | ENSG00000074181 | NOTCH3 | 13 | 37.14% | notch receptor 3 [Source:HGNC Symbol;Acc:HGNC:7883] |
| CHD3 | ENSG00000170004 | CHD3 | 13 | 37.14% | chromodomain helicase DNA binding protein 3 [Source:HGNC Symbol;Acc:HGNC:1918] |
| CPAMD8 | ENSG00000160111 | CPAMD8 | 12 | 34.29% | C3 and PZP like alpha-2-macroglobulin domain containing 8 [Source:HGNC Symbol;Acc:HGNC:23228] |
| RTN1 | ENSG00000139970 | RTN1 | 11 | 31.43% | reticulon 1 [Source:HGNC Symbol;Acc:HGNC:10467] |
| PCNT | ENSG00000160299 | PCNT | 11 | 31.43% | pericentrin [Source:HGNC Symbol;Acc:HGNC:16068] |
| VEGFA | ENSG00000112715 | VEGFA | 11 | 31.43% | vascular endothelial growth factor A [Source:HGNC Symbol;Acc:HGNC:12680] |
| PODN | ENSG00000174348 | PODN | 10 | 28.57% | podocan [Source:HGNC Symbol;Acc:HGNC:23174] |
| MAP3K4 | ENSG00000085511 | MAP3K4 | 10 | 28.57% | mitogen-activated protein kinase kinase kinase 4 [Source:HGNC Symbol;Acc:HGNC:6856] |
| MAML1 | ENSG00000161021 | MAML1 | 9 | 25.71% | mastermind like transcriptional coactivator 1 [Source:HGNC Symbol;Acc:HGNC:13632] |
| E2F5 | ENSG00000133740 | E2F5 | 9 | 25.71% | E2F transcription factor 5 [Source:HGNC Symbol;Acc:HGNC:3119] |
| FOXE1 | ENSG00000178919 | FOXE1 | 9 | 25.71% | forkhead box E1 [Source:HGNC Symbol;Acc:HGNC:3806] |
| GRIK4 | ENSG00000149403 | GRIK4 | 9 | 25.71% | glutamate ionotropic receptor kainate type subunit 4 [Source:HGNC Symbol;Acc:HGNC:4582] |
| HNRNPUL2 | ENSG00000214753 | HNRNPUL2 | 8 | 22.86% | heterogeneous nuclear ribonucleoprotein U like 2 [Source:HGNC Symbol;Acc:HGNC:25451] |
| HES7 | ENSG00000179111 | HES7 | 8 | 22.86% | hes family bHLH transcription factor 7 [Source:HGNC Symbol;Acc:HGNC:15977] |
| SPTBN5 | ENSG00000137877 | SPTBN5 | 8 | 22.86% | spectrin beta, non-erythrocytic 5 [Source:HGNC Symbol;Acc:HGNC:15680] |
| MDC1 | ENSG00000137337 | MDC1 | 7 | 20% | mediator of DNA damage checkpoint 1 [Source:HGNC Symbol;Acc:HGNC:21163] |
| CT45A5 | ENSG00000228836 | CT45A5 | 7 | 20% | cancer/testis antigen family 45 member A5 [Source:HGNC Symbol;Acc:HGNC:33270] |
| FIZ1 | ENSG00000179943 | FIZ1 | 7 | 20% | FLT3 interacting zinc finger 1 [Source:HGNC Symbol;Acc:HGNC:25917] |
| DNAH9 | ENSG00000007174 | DNAH9 | 7 | 20% | dynein axonemal heavy chain 9 [Source:HGNC Symbol;Acc:HGNC:2953] |
| HMGA2 | ENSG00000149948 | HMGA2 | 7 | 20% | high mobility group AT-hook 2 [Source:HGNC Symbol;Acc:HGNC:5009] |
| MMP28 | ENSG00000271447 | MMP28 | 7 | 20% | matrix metallopeptidase 28 [Source:HGNC Symbol;Acc:HGNC:14366] |
| MAPK3 | ENSG00000102882 | MAPK3 | 7 | 20% | mitogen-activated protein kinase 3 [Source:HGNC Symbol;Acc:HGNC:6877] |
| DLL3 | ENSG00000090932 | DLL3 | 7 | 20% | delta like canonical Notch ligand 3 [Source:HGNC Symbol;Acc:HGNC:2909] |
