# Supplementary material for: Exploring Somatic Alteration Associating With Aggressive Behaviors of Papillary Thyroid Carcinomas by Targeted Sequencing
Source: Front Oncol. 2021 Oct 7;11:722814. doi: 10.3389/fonc.2021.722814 (PMC8529196; doi:10.3389/fonc.2021.722814)
Supplement: Supplementary file 6 [file Table_4.docx]

**Table S4 List of enriched pathways from IPA**

| **Ingenuity Canonical Pathways** | **-log(p-value)** | **p-value** | **Ratio** | **Molecules** |
| --- | --- | --- | --- | --- |
| Notch Signaling | 6.69 | 2.04174E-07 | 0.108 | DLL3,HES7,MAML1,NOTCH3 |
| Tumor Microenvironment Pathway | 5.36 | 4.36516E-06 | 0.0284 | BRAF,HLA-G,MAPK3,MMP28,VEGFA |
| Regulation of the Epithelial-Mesenchymal Transition Pathway | 5.17 | 6.76083E-06 | 0.026 | BRAF,HMGA2,MAML1,MAPK3,NOTCH3 |
| HIF1α Signaling | 3.73 | 0.000186209 | 0.0195 | BRAF,MAPK3,MMP28,VEGFA |
| Bladder Cancer Signaling | 3.47 | 0.000338844 | 0.0309 | MAPK3,MMP28,VEGFA |
| Colorectal Cancer Metastasis Signaling | 3.38 | 0.000416869 | 0.0158 | BRAF,MAPK3,MMP28,VEGFA |
| Pancreatic Adenocarcinoma Signaling | 3.32 | 0.00047863 | 0.0275 | E2F5,MAPK3,VEGFA |
| MSP-RON Signaling In Cancer Cells Pathway | 3.06 | 0.000870964 | 0.0224 | BRAF,MAPK3,VEGFA |
| Ovarian Cancer Signaling | 3.02 | 0.000954993 | 0.0216 | BRAF,MAPK3,VEGFA |
| Corticotropin Releasing Hormone Signaling | 2.96 | 0.001096478 | 0.0207 | BRAF,MAPK3,VEGFA |
| Estrogen Receptor Signaling | 2.96 | 0.001096478 | 0.0122 | MAPK3,MMP28,NCOR2,VEGFA |
| Relaxin Signaling | 2.91 | 0.001230269 | 0.0199 | BRAF,MAPK3,VEGFA |
| T Cell Exhaustion Signaling Pathway | 2.73 | 0.001862087 | 0.0171 | HLA-G,MAPK3,VEGFA |
| Melanoma Signaling | 2.65 | 0.002238721 | 0.04 | BRAF,MAPK3 |
| IL-17 Signaling | 2.65 | 0.002238721 | 0.016 | MAPK3,MUC5B,VEGFA |
| Regulation Of The Epithelial Mesenchymal Transition By Growth Factors Pathway | 2.64 | 0.002290868 | 0.016 | BRAF,HMGA2,MAPK3 |
| UVC-Induced MAPK Signaling | 2.63 | 0.002344229 | 0.0392 | BRAF,MAPK3 |
| Natural Killer Cell Signaling | 2.58 | 0.002630268 | 0.0152 | HLA-G,MAP3K4,MAPK3 |
| IL-8 Signaling | 2.56 | 0.002754229 | 0.015 | BRAF,MAPK3,VEGFA |
| S-methyl-5-thio-α-D-ribose 1-phosphate Degradation | 2.55 | 0.002818383 | 0.5 | MRI1 |
| Role of CHK Proteins in Cell Cycle Checkpoint Control | 2.54 | 0.002884032 | 0.0351 | E2F5,MDC1 |
| Cancer Drug Resistance By Drug Efflux | 2.52 | 0.003019952 | 0.0345 | BRAF,MAPK3 |
| IL-17A Signaling in Airway Cells | 2.43 | 0.003715352 | 0.0308 | MAPK3,MUC5B |
| Pyridoxal 5'-phosphate Salvage Pathway | 2.41 | 0.003890451 | 0.0303 | BRAF,MAPK3 |
| Melatonin Signaling | 2.34 | 0.004570882 | 0.0278 | BRAF,MAPK3 |
| Role of MAPK Signaling in Inhibiting the Pathogenesis of Influenza | 2.3 | 0.005011872 | 0.0267 | BRAF,MAPK3 |
| Antiproliferative Role of Somatostatin Receptor 2 | 2.28 | 0.005248075 | 0.026 | BRAF,MAPK3 |
| Thyroid Cancer Signaling | 2.26 | 0.005495409 | 0.0253 | BRAF,MAPK3 |
| Role of BRCA1 in DNA Damage Response | 2.25 | 0.005623413 | 0.025 | E2F5,MDC1 |
| Renal Cell Carcinoma Signaling | 2.25 | 0.005623413 | 0.025 | MAPK3,VEGFA |
| VEGF Family Ligand-Receptor Interactions | 2.21 | 0.00616595 | 0.0238 | MAPK3,VEGFA |
| Senescence Pathway | 2.18 | 0.006606934 | 0.0109 | BRAF,E2F5,MAPK3 |
| Acute Myeloid Leukemia Signaling | 2.16 | 0.00691831 | 0.0225 | BRAF,MAPK3 |
| RANK Signaling in Osteoclasts | 2.16 | 0.00691831 | 0.0225 | MAP3K4,MAPK3 |
| Xenobiotic Metabolism Signaling | 2.13 | 0.007413102 | 0.0105 | MAP3K4,MAPK3,NCOR2 |
| Amyotrophic Lateral Sclerosis Signaling | 2.09 | 0.008128305 | 0.0206 | GRIK4,VEGFA |
| Salvage Pathways of Pyrimidine Ribonucleotides | 2.08 | 0.008317638 | 0.0204 | BRAF,MAPK3 |
| Nitric Oxide Signaling in the Cardiovascular System | 2.07 | 0.00851138 | 0.0202 | MAPK3,VEGFA |
| VEGF Signaling | 2.07 | 0.00851138 | 0.0202 | MAPK3,VEGFA |
| Chronic Myeloid Leukemia Signaling | 2.04 | 0.009120108 | 0.0194 | E2F5,MAPK3 |
| PPAR Signaling | 2.02 | 0.009549926 | 0.019 | MAPK3,NCOR2 |
| Gαs Signaling | 2.01 | 0.009772372 | 0.0187 | BRAF,MAPK3 |
| Glioma Signaling | 1.98 | 0.010471285 | 0.0182 | E2F5,MAPK3 |
| Role of MAPK Signaling in Promoting the Pathogenesis of Influenza | 1.98 | 0.010471285 | 0.0182 | BRAF,MAPK3 |
| NGF Signaling | 1.95 | 0.011220185 | 0.0175 | MAP3K4,MAPK3 |
| Endocannabinoid Developing Neuron Pathway | 1.95 | 0.011220185 | 0.0174 | BRAF,MAPK3 |
| Role of Tissue Factor in Cancer | 1.94 | 0.011481536 | 0.0172 | MAPK3,VEGFA |
| HGF Signaling | 1.91 | 0.012302688 | 0.0167 | MAP3K4,MAPK3 |
| IL-6 Signaling | 1.87 | 0.013489629 | 0.0159 | MAPK3,VEGFA |
| Ferroptosis Signaling Pathway | 1.87 | 0.013489629 | 0.0159 | BRAF,MAPK3 |
| STAT3 Pathway | 1.82 | 0.015135612 | 0.0148 | MAPK3,VEGFA |
| Hepatic Fibrosis Signaling Pathway | 1.8 | 0.015848932 | 0.00794 | BRAF,MAPK3,VEGFA |
| Aryl Hydrocarbon Receptor Signaling | 1.77 | 0.016982437 | 0.014 | MAPK3,NCOR2 |
| Endocannabinoid Cancer Inhibition Pathway | 1.77 | 0.016982437 | 0.014 | MAPK3,VEGFA |
| Xenobiotic Metabolism General Signaling Pathway | 1.77 | 0.016982437 | 0.014 | MAP3K4,MAPK3 |
| Molecular Mechanisms of Cancer | 1.74 | 0.018197009 | 0.0075 | BRAF,E2F5,MAPK3 |
| Coronavirus Pathogenesis Pathway | 1.73 | 0.018620871 | 0.0133 | E2F5,MAPK3 |
| PKCθ Signaling in T Lymphocytes | 1.7 | 0.019952623 | 0.0129 | MAP3K4,MAPK3 |
| Glioblastoma Multiforme Signaling | 1.65 | 0.022387211 | 0.0121 | E2F5,MAPK3 |
| Germ Cell-Sertoli Cell Junction Signaling | 1.62 | 0.023988329 | 0.0117 | MAP3K4,MAPK3 |
| GNRH Signaling | 1.61 | 0.024547089 | 0.0116 | MAP3K4,MAPK3 |
| GADD45 Signaling | 1.58 | 0.02630268 | 0.0526 | MAP3K4 |
| B Cell Receptor Signaling | 1.55 | 0.028183829 | 0.0108 | MAP3K4,MAPK3 |
| Endothelin-1 Signaling | 1.55 | 0.028183829 | 0.0106 | BRAF,MAPK3 |
| Production of Nitric Oxide and Reactive Oxygen Species in Macrophages | 1.54 | 0.028840315 | 0.0106 | MAP3K4,MAPK3 |
| Ephrin Receptor Signaling | 1.54 | 0.028840315 | 0.0106 | MAPK3,VEGFA |
| ILK Signaling | 1.54 | 0.028840315 | 0.0105 | MAPK3,VEGFA |
| PPARα/RXRα Activation | 1.53 | 0.029512092 | 0.0105 | MAPK3,NCOR2 |
| Leukocyte Extravasation Signaling | 1.52 | 0.030199517 | 0.0104 | MAP3K4,MMP28 |
| RAR Activation | 1.52 | 0.030199517 | 0.0103 | NCOR2,VEGFA |
| HER-2 Signaling in Breast Cancer | 1.52 | 0.030199517 | 0.0103 | BRAF,MAPK3 |
| Sertoli Cell-Sertoli Cell Junction Signaling | 1.52 | 0.030199517 | 0.0103 | MAP3K4,MAPK3 |
| Adrenomedullin signaling pathway | 1.51 | 0.030902954 | 0.0102 | BRAF,MAPK3 |
| Axonal Guidance Signaling | 1.5 | 0.031622777 | 0.00607 | MAPK3,MMP28,VEGFA |
| ERK/MAPK Signaling | 1.49 | 0.032359366 | 0.0099 | BRAF,MAPK3 |
| IL-22 Signaling | 1.48 | 0.033113112 | 0.0417 | MAPK3 |
| Role of JAK family kinases in IL-6-type Cytokine Signaling | 1.46 | 0.034673685 | 0.04 | MAPK3 |
| mTOR Signaling | 1.46 | 0.034673685 | 0.00952 | MAPK3,VEGFA |
| Integrin Signaling | 1.45 | 0.035481339 | 0.00939 | BRAF,MAPK3 |
| IL-17A Signaling in Gastric Cells | 1.44 | 0.036307805 | 0.0385 | MAPK3 |
| Estrogen-mediated S-phase Entry | 1.44 | 0.036307805 | 0.0385 | E2F5 |
| EIF2 Signaling | 1.41 | 0.038904514 | 0.00893 | MAPK3,VEGFA |
| Systemic Lupus Erythematosus Signaling | 1.39 | 0.040738028 | 0.00873 | HLA-G,MAPK3 |
| cAMP-mediated signaling | 1.39 | 0.040738028 | 0.00873 | BRAF,MAPK3 |
| 4-1BB Signaling in T Lymphocytes | 1.36 | 0.043651583 | 0.0312 | MAPK3 |
| Huntington's Disease Signaling | 1.36 | 0.043651583 | 0.00837 | MAPK3,NCOR2 |
| Cardiac Hypertrophy Signaling | 1.35 | 0.044668359 | 0.00833 | MAP3K4,MAPK3 |
| Inhibition of Angiogenesis by TSP1 | 1.33 | 0.046773514 | 0.0294 | VEGFA |
| MIF-mediated Glucocorticoid Regulation | 1.33 | 0.046773514 | 0.0294 | MAPK3 |
| Opioid Signaling Pathway | 1.33 | 0.046773514 | 0.0081 | BRAF,MAPK3 |
| DNA Methylation and Transcriptional Repression Signaling | 1.32 | 0.047863009 | 0.0286 | CHD3 |
| Breast Cancer Regulation by Stathmin1 | 1.31 | 0.048977882 | 0.00508 | E2F5,MAPK3,VEGFA |
| IL-17A Signaling in Fibroblasts | 1.31 | 0.048977882 | 0.0278 | MAPK3 |
| CREB Signaling in Neurons | 1.3 | 0.050118723 | 0.00503 | GRIK4,MAPK3,VEGFA |
| Cell Cycle Regulation by BTG Family Proteins | 1.3 | 0.050118723 | 0.027 | E2F5 |
| Inhibition of Matrix Metalloproteases | 1.27 | 0.05370318 | 0.0256 | MMP28 |
| Antigen Presentation Pathway | 1.27 | 0.05370318 | 0.0256 | HLA-G |
| G-Protein Coupled Receptor Signaling | 1.25 | 0.056234133 | 0.0073 | BRAF,MAPK3 |
| MIF Regulation of Innate Immunity | 1.24 | 0.057543994 | 0.0238 | MAPK3 |
| Oncostatin M Signaling | 1.23 | 0.058884366 | 0.0233 | MAPK3 |
| Role of IL-17F in Allergic Inflammatory Airway Diseases | 1.23 | 0.058884366 | 0.0233 | MAPK3 |
| BAG2 Signaling Pathway | 1.23 | 0.058884366 | 0.0233 | MAPK3 |
| PFKFB4 Signaling Pathway | 1.2 | 0.063095734 | 0.0217 | MAPK3 |
| Graft-versus-Host Disease Signaling | 1.19 | 0.064565423 | 0.0208 | HLA-G |
| Autoimmune Thyroid Disease Signaling | 1.18 | 0.066069345 | 0.0204 | HLA-G |
| Amyloid Processing | 1.17 | 0.067608298 | 0.02 | MAPK3 |
| UVB-Induced MAPK Signaling | 1.15 | 0.070794578 | 0.0192 | MAPK3 |
| Synaptogenesis Signaling Pathway | 1.15 | 0.070794578 | 0.00641 | BRAF,MAPK3 |
| Role of Macrophages, Fibroblasts and Endothelial Cells in Rheumatoid Arthritis | 1.15 | 0.070794578 | 0.00637 | MAPK3,VEGFA |
| CD27 Signaling in Lymphocytes | 1.14 | 0.072443596 | 0.0189 | MAP3K4 |
| Lymphotoxin β Receptor Signaling | 1.14 | 0.072443596 | 0.0189 | MAPK3 |
| Role of IL-17A in Arthritis | 1.13 | 0.074131024 | 0.0182 | MAPK3 |
| EGF Signaling | 1.13 | 0.074131024 | 0.0182 | MAPK3 |
| CNTF Signaling | 1.11 | 0.077624712 | 0.0175 | MAPK3 |
| Glutamate Receptor Signaling | 1.11 | 0.077624712 | 0.0175 | GRIK4 |
| Systemic Lupus Erythematosus In T Cell Signaling Pathway | 1.1 | 0.079432823 | 0.00599 | HLA-G,MAPK3 |
| Semaphorin Signaling in Neurons | 1.09 | 0.081283052 | 0.0167 | MAPK3 |
| Endometrial Cancer Signaling | 1.09 | 0.081283052 | 0.0167 | MAPK3 |
| IL-2 Signaling | 1.09 | 0.081283052 | 0.0164 | MAPK3 |
| Thrombopoietin Signaling | 1.07 | 0.085113804 | 0.0159 | MAPK3 |
| Role of PI3K/AKT Signaling in the Pathogenesis of Influenza | 1.07 | 0.085113804 | 0.0156 | MAPK3 |
| CD40 Signaling | 1.06 | 0.087096359 | 0.0154 | MAPK3 |
| ErbB2-ErbB3 Signaling | 1.06 | 0.087096359 | 0.0154 | MAPK3 |
| ErbB4 Signaling | 1.05 | 0.089125094 | 0.0149 | MAPK3 |
| Cell Cycle: G1/S Checkpoint Regulation | 1.05 | 0.089125094 | 0.0149 | E2F5 |
| Nur77 Signaling in T Lymphocytes | 1.04 | 0.091201084 | 0.0147 | MAPK3 |
| Role of JAK1 and JAK3 in γc Cytokine Signaling | 1.03 | 0.09332543 | 0.0145 | MAPK3 |
| SPINK1 General Cancer Pathway | 1.03 | 0.09332543 | 0.0145 | MAPK3 |
| Agrin Interactions at Neuromuscular Junction | 1.03 | 0.09332543 | 0.0143 | MAPK3 |
| GM-CSF Signaling | 1.03 | 0.09332543 | 0.0143 | MAPK3 |
| Growth Hormone Signaling | 1.02 | 0.095499259 | 0.0141 | MAPK3 |
| Ephrin B Signaling | 1.02 | 0.095499259 | 0.0139 | MAPK3 |
| Non-Small Cell Lung Cancer Signaling | 1.01 | 0.097723722 | 0.0137 | MAPK3 |
| Glioma Invasiveness Signaling | 1.01 | 0.097723722 | 0.0137 | MAPK3 |
| Leptin Signaling in Obesity | 1.01 | 0.097723722 | 0.0135 | MAPK3 |
| Regulation of Cellular Mechanics by Calpain Protease | 1.01 | 0.097723722 | 0.0135 | MAPK3 |
| Hypoxia Signaling in the Cardiovascular System | 1.01 | 0.097723722 | 0.0135 | VEGFA |
| TREM1 Signaling | 1 | 0.1 | 0.0133 | MAPK3 |
| IL-15 Signaling | 1 | 0.1 | 0.0133 | MAPK3 |
| Estrogen-Dependent Breast Cancer Signaling | 1 | 0.1 | 0.0133 | MAPK3 |
| NF-κB Activation by Viruses | 0.996 | 0.100925289 | 0.0132 | MAPK3 |
| GDNF Family Ligand-Receptor Interactions | 0.996 | 0.100925289 | 0.0132 | MAPK3 |
| Neurotrophin/TRK Signaling | 0.996 | 0.100925289 | 0.0132 | MAPK3 |
| VDR/RXR Activation | 0.983 | 0.103992017 | 0.0128 | NCOR2 |
| IL-7 Signaling Pathway | 0.983 | 0.103992017 | 0.0128 | MAPK3 |
| IL-3 Signaling | 0.979 | 0.104954243 | 0.0127 | MAPK3 |
| BEX2 Signaling Pathway | 0.979 | 0.104954243 | 0.0127 | VEGFA |
| FLT3 Signaling in Hematopoietic Progenitor Cells | 0.975 | 0.105925373 | 0.0125 | MAPK3 |
| Role of MAPK Signaling in the Pathogenesis of Influenza | 0.975 | 0.105925373 | 0.0125 | MAPK3 |
| JAK/Stat Signaling | 0.975 | 0.105925373 | 0.0125 | MAPK3 |
| Chemokine Signaling | 0.975 | 0.105925373 | 0.0125 | MAPK3 |
| Prolactin Signaling | 0.967 | 0.107894672 | 0.0123 | MAPK3 |
| Protein Kinase A Signaling | 0.967 | 0.107894672 | 0.005 | BRAF,MAPK3 |
| Cyclins and Cell Cycle Regulation | 0.967 | 0.107894672 | 0.0123 | E2F5 |
| PEDF Signaling | 0.963 | 0.108893009 | 0.0122 | MAPK3 |
| Apelin Adipocyte Signaling Pathway | 0.963 | 0.108893009 | 0.0122 | MAPK3 |
| LPS-stimulated MAPK Signaling | 0.959 | 0.109900584 | 0.012 | MAPK3 |
| TR/RXR Activation | 0.955 | 0.110917482 | 0.0119 | NCOR2 |
| FGF Signaling | 0.955 | 0.110917482 | 0.0119 | MAPK3 |
| Regulation Of The Epithelial Mesenchymal Transition In Development Pathway | 0.955 | 0.110917482 | 0.0119 | MAML1 |
| BMP signaling pathway | 0.947 | 0.112979591 | 0.0118 | MAPK3 |
| Allograft Rejection Signaling | 0.943 | 0.114024979 | 0.0116 | HLA-G |
| PDGF Signaling | 0.943 | 0.114024979 | 0.0116 | MAPK3 |
| Ceramide Signaling | 0.936 | 0.115877736 | 0.0114 | MAPK3 |
| Crosstalk between Dendritic Cells and Natural Killer Cells | 0.928 | 0.118032064 | 0.0112 | HLA-G |
| Regulation of IL-2 Expression in Activated and Anergic T Lymphocytes | 0.924 | 0.119124201 | 0.0111 | MAPK3 |
| OX40 Signaling Pathway | 0.924 | 0.119124201 | 0.0111 | HLA-G |
| Prostate Cancer Signaling | 0.921 | 0.11994993 | 0.011 | MAPK3 |
| Fcγ Receptor-mediated Phagocytosis in Macrophages and Monocytes | 0.907 | 0.123879659 | 0.0106 | MAPK3 |
| Melanocyte Development and Pigmentation Signaling | 0.907 | 0.123879659 | 0.0106 | MAPK3 |
| ErbB Signaling | 0.907 | 0.123879659 | 0.0106 | MAPK3 |
| α-Adrenergic Signaling | 0.903 | 0.125025903 | 0.0105 | MAPK3 |
| Communication between Innate and Adaptive Immune Cells | 0.9 | 0.125892541 | 0.0104 | HLA-G |
| TGF-β Signaling | 0.9 | 0.125892541 | 0.0104 | MAPK3 |
| ATM Signaling | 0.893 | 0.12793813 | 0.0103 | MDC1 |
| UVA-Induced MAPK Signaling | 0.889 | 0.129121927 | 0.0102 | MAPK3 |
| Apelin Cardiomyocyte Signaling Pathway | 0.886 | 0.130016958 | 0.0101 | MAPK3 |
| Apoptosis Signaling | 0.883 | 0.130918192 | 0.01 | MAPK3 |
| Neuropathic Pain Signaling In Dorsal Horn Neurons | 0.879 | 0.132129563 | 0.0099 | MAPK3 |
| SAPK/JNK Signaling | 0.873 | 0.133967669 | 0.0098 | MAP3K4 |
| Mouse Embryonic Stem Cell Pluripotency | 0.87 | 0.134896288 | 0.00971 | MAPK3 |
| FAK Signaling | 0.866 | 0.136144468 | 0.00962 | MAPK3 |
| IGF-1 Signaling | 0.866 | 0.136144468 | 0.00962 | MAPK3 |
| Glucocorticoid Receptor Signaling | 0.863 | 0.137088177 | 0.00433 | MAPK3,NCOR2 |
| Neuregulin Signaling | 0.863 | 0.137088177 | 0.00952 | MAPK3 |
| PAK Signaling | 0.857 | 0.138995263 | 0.00943 | MAPK3 |
| T Cell Receptor Signaling | 0.857 | 0.138995263 | 0.00943 | MAPK3 |
| PD-1, PD-L1 cancer immunotherapy pathway | 0.857 | 0.138995263 | 0.00943 | HLA-G |
| Telomerase Signaling | 0.854 | 0.139958732 | 0.00935 | MAPK3 |
| CDK5 Signaling | 0.851 | 0.14092888 | 0.00926 | MAPK3 |
| Antioxidant Action of Vitamin C | 0.848 | 0.141905752 | 0.00917 | MAPK3 |
| Type I Diabetes Mellitus Signaling | 0.839 | 0.144877185 | 0.00901 | HLA-G |
| MSP-RON Signaling In Macrophages Pathway | 0.833 | 0.146892628 | 0.00885 | MAPK3 |
| Apelin Endothelial Signaling Pathway | 0.827 | 0.148936108 | 0.0087 | MAPK3 |
| fMLP Signaling in Neutrophils | 0.821 | 0.151008015 | 0.00862 | MAPK3 |
| Neuroprotective Role of THOP1 in Alzheimer's Disease | 0.821 | 0.151008015 | 0.00862 | HLA-G |
| Fc Epsilon RI Signaling | 0.818 | 0.152054753 | 0.00855 | MAPK3 |
| Sphingosine-1-phosphate Signaling | 0.818 | 0.152054753 | 0.00855 | MAPK3 |
| Role of PKR in Interferon Induction and Antiviral Response | 0.815 | 0.153108746 | 0.00847 | MAPK3 |
| Renin-Angiotensin Signaling | 0.815 | 0.153108746 | 0.00847 | MAPK3 |
| Cholecystokinin/Gastrin-mediated Signaling | 0.812 | 0.154170045 | 0.0084 | MAPK3 |
| Role of NANOG in Mammalian Embryonic Stem Cell Pluripotency | 0.812 | 0.154170045 | 0.0084 | MAPK3 |
| Cardiac Hypertrophy Signaling (Enhanced) | 0.812 | 0.154170045 | 0.00402 | MAP3K4,MAPK3 |
| LXR/RXR Activation | 0.804 | 0.15703628 | 0.00826 | NCOR2 |
| Rac Signaling | 0.804 | 0.15703628 | 0.00826 | MAPK3 |
| Th1 Pathway | 0.804 | 0.15703628 | 0.00826 | NOTCH3 |
| Reelin Signaling in Neurons | 0.801 | 0.158124804 | 0.0082 | MAPK3 |
| G Beta Gamma Signaling | 0.801 | 0.158124804 | 0.0082 | MAPK3 |
| Inhibition of ARE-Mediated mRNA Degradation Pathway | 0.801 | 0.158124804 | 0.0082 | MAPK3 |
| CCR3 Signaling in Eosinophils | 0.796 | 0.159955803 | 0.00806 | MAPK3 |
| Gαi Signaling | 0.793 | 0.161064564 | 0.008 | MAPK3 |
| 14-3-3-mediated Signaling | 0.785 | 0.164058977 | 0.00787 | MAPK3 |
| P2Y Purigenic Receptor Signaling Pathway | 0.785 | 0.164058977 | 0.00787 | MAPK3 |
| Endocannabinoid Neuronal Synapse Pathway | 0.783 | 0.164816239 | 0.00781 | MAPK3 |
| Synaptic Long Term Potentiation | 0.78 | 0.165958691 | 0.00775 | MAPK3 |
| p70S6K Signaling | 0.78 | 0.165958691 | 0.00775 | MAPK3 |
| White Adipose Tissue Browning Pathway | 0.78 | 0.165958691 | 0.00775 | VEGFA |
| Gα12/13 Signaling | 0.775 | 0.167880402 | 0.00763 | MAPK3 |
| IL-12 Signaling and Production in Macrophages | 0.767 | 0.171001532 | 0.00752 | MAPK3 |
| Androgen Signaling | 0.759 | 0.174180687 | 0.00735 | MAPK3 |
| PTEN Signaling | 0.759 | 0.174180687 | 0.00735 | MAPK3 |
| Th2 Pathway | 0.759 | 0.174180687 | 0.00735 | NOTCH3 |
| Iron homeostasis signaling pathway | 0.757 | 0.174984669 | 0.0073 | MAPK3 |
| PI3K Signaling in B Lymphocytes | 0.752 | 0.177010896 | 0.00725 | MAPK3 |
| Insulin Receptor Signaling | 0.75 | 0.177827941 | 0.00719 | MAPK3 |
| Type II Diabetes Mellitus Signaling | 0.742 | 0.181134009 | 0.00704 | MAPK3 |
| Epithelial Adherens Junction Signaling | 0.714 | 0.193196832 | 0.00658 | NOTCH3 |
| Role of Pattern Recognition Receptors in Recognition of Bacteria and Viruses | 0.71 | 0.19498446 | 0.00649 | MAPK3 |
| Gαq Signaling | 0.703 | 0.198152703 | 0.00637 | MAPK3 |
| Aldosterone Signaling in Epithelial Cells | 0.701 | 0.199067334 | 0.00633 | MAPK3 |
| eNOS Signaling | 0.697 | 0.200909281 | 0.00629 | VEGFA |
| HOTAIR Regulatory Pathway | 0.695 | 0.201836636 | 0.00625 | MMP28 |
| HMGB1 Signaling | 0.684 | 0.207014135 | 0.00606 | MAPK3 |
| Regulation of eIF4 and p70S6K Signaling | 0.682 | 0.207969669 | 0.00602 | MAPK3 |
| CXCR4 Signaling | 0.678 | 0.209893988 | 0.00599 | MAPK3 |
| Th1 and Th2 Activation Pathway | 0.67 | 0.213796209 | 0.00585 | NOTCH3 |
| Erythropoietin Signaling Pathway | 0.666 | 0.215774441 | 0.00578 | MAPK3 |
| Granulocyte Adhesion and Diapedesis | 0.666 | 0.215774441 | 0.00578 | MMP28 |
| Cdc42 Signaling | 0.658 | 0.219785987 | 0.00568 | HLA-G |
| NF-κB Signaling | 0.652 | 0.222843515 | 0.00559 | BRAF |
| Acute Phase Response Signaling | 0.65 | 0.223872114 | 0.00556 | MAPK3 |
| Role of NFAT in Regulation of the Immune Response | 0.648 | 0.224905461 | 0.00552 | MAPK3 |
| Dendritic Cell Maturation | 0.642 | 0.228034207 | 0.00543 | MAPK3 |
| PI3K/AKT Signaling | 0.642 | 0.228034207 | 0.00543 | MAPK3 |
| Hepatic Cholestasis | 0.636 | 0.231206479 | 0.00538 | MAP3K4 |
| Hepatic Fibrosis / Hepatic Stellate Cell Activation | 0.636 | 0.231206479 | 0.00538 | VEGFA |
| Synaptic Long Term Depression | 0.631 | 0.233883724 | 0.00529 | MAPK3 |
| NRF2-mediated Oxidative Stress Response | 0.631 | 0.233883724 | 0.00529 | MAPK3 |
| Xenobiotic Metabolism CAR Signaling Pathway | 0.631 | 0.233883724 | 0.00529 | MAPK3 |
| Xenobiotic Metabolism PXR Signaling Pathway | 0.625 | 0.237137371 | 0.00521 | NCOR2 |
| Clathrin-mediated Endocytosis Signaling | 0.623 | 0.238231947 | 0.00518 | VEGFA |
| Agranulocyte Adhesion and Diapedesis | 0.623 | 0.238231947 | 0.00518 | MMP28 |
| Gap Junction Signaling | 0.613 | 0.243781082 | 0.00505 | MAPK3 |
| Calcium Signaling | 0.599 | 0.251767693 | 0.00485 | MAPK3 |
| Thrombin Signaling | 0.595 | 0.254097271 | 0.00481 | MAPK3 |
| Role of NFAT in Cardiac Hypertrophy | 0.585 | 0.260015956 | 0.00467 | MAPK3 |
| Role of Osteoblasts, Osteoclasts and Chondrocytes in Rheumatoid Arthritis | 0.577 | 0.264850014 | 0.00459 | MAPK3 |
| Osteoarthritis Pathway | 0.573 | 0.267300641 | 0.00455 | VEGFA |
| Actin Cytoskeleton Signaling | 0.562 | 0.274157417 | 0.00441 | MAPK3 |
| Insulin Secretion Signaling Pathway | 0.536 | 0.291071712 | 0.0041 | MAPK3 |
| Signaling by Rho Family GTPases | 0.523 | 0.299916252 | 0.00395 | MAPK3 |
| Phospholipase C Signaling | 0.504 | 0.313328572 | 0.00376 | MAPK3 |
| Systemic Lupus Erythematosus In B Cell Signaling Pathway | 0.492 | 0.322106879 | 0.00364 | MAPK3 |
| Sirtuin Signaling Pathway | 0.472 | 0.337287309 | 0.00344 | MAPK3 |
| Neuroinflammation Signaling Pathway | 0.462 | 0.345143739 | 0.00333 | MAPK3 |
| **Ingenuity Canonical Pathways** | **-log(p-value)** | **p-value** | **Ratio** | **Molecules** |
