# Supplementary material for: Exploring Somatic Alteration Associating With Aggressive Behaviors of Papillary Thyroid Carcinomas by Targeted Sequencing
Source: Front Oncol. 2021 Oct 7;11:722814. doi: 10.3389/fonc.2021.722814 (PMC8529196; doi:10.3389/fonc.2021.722814)
Supplement: Supplementary file 7 [file Table_5.docx]

**Table S5 Quantification of interesting gene expression in IHC**

|  | MDC1 | BRAF | RBL2 | NCOR2 |
| --- | --- | --- | --- | --- |
| Normal | 0.077±0.008 | 0.050±0.006 | 0.031±0.005 | 0.028±0.001 |
| Aggressive PTC | 0.434±0.057 | 0.226±0.018 | 0.215±0.016 | 0.198±0.021 |
| Mild PTC | 0.177±0.021 | 0.136±0.011 | 0.206±0.014 | 0.154±0.012 |

The numbers were calculated as percentage of average IOD (integrated optical density/area)
